# Activation of synovial fibroblasts from patients at revision of their metal-on-metal total hip arthroplasty

**DOI:** 10.1186/s12989-020-00374-y

**Published:** 2020-08-27

**Authors:** Jing Xu, Junyao Yang, Jian Chen, Xiaoli Zhang, Yuanhao Wu, Alister Hart, Agata Nyga, Julia C. Shelton

**Affiliations:** 1 Department of Paediatric Orthopaedics, Xinhua Hospital, School of Medicine, Shanghai Jiao Tong University, Shanghai, 200092 China; 2 Institute of Bioengineering, School of Engineering and Materials Science, Queen Mary University of London, London, UK; 3 Cardiovascular Division, Faculty of Life Science and Medicine, King’s College London, London, SE5 9NU UK; 4 Department of Spine Surgery, Xinhua Hospital, School of Medicine, Shanghai Jiao Tong University, Shanghai, 200092 China; 5 Institute of Orthopaedics & Musculoskeletal Science, Royal National Orthopaedic Hospital, University College London, Stanmore, HA7 4AP UK; 6 Research Department of Surgical Biotechnology, Division of Surgery and Interventional Sciences, University College London, London, NW3 2QG UK; 7 Current affiliation: MRC LMB, Cambridge Biomedical Campus, Francis Crick Avenue, Cambridge, CB2 0QH UK

**Keywords:** Cobalt chromium debris, Synovial fibroblast, Inflammation, Fibrosis

## Abstract

**Background:**

The toxicity of released metallic particles generated in metal-on-metal (MoM) total hip arthroplasty (THA) using cobalt chromium (CoCr) has raised concerns regarding their safety amongst both surgeons and the public. Soft tissue changes such as pseudotumours and metallosis have been widely observed following the use of these implants, which release metallic by-products due to both wear and corrosion. Although activated fibroblasts, the dominant cell type in soft tissues, have been linked to many diseases, the role of synovial fibroblasts in the adverse reactions caused by CoCr implants remains unknown. To investigate the influence of implants manufactured from CoCr, the periprosthetic synovial tissues and synovial fibroblasts from patients with failed MoM THA, undergoing a revision operation, were analysed and compared with samples from patients undergoing a primary hip replacement, in order to elucidate histological and cellular changes.

**Results:**

Periprosthetic tissue from patients with MoM implants was characterized by marked fibrotic changes, notably an increase in collagen content from less than 20% to 45–55%, an increase in α-smooth muscle actin positive cells from 4 to 9% as well as immune cells infiltration. Primary cell culture results demonstrated that MoM synovial fibroblasts have a decreased apoptosis rate from 14 to 6% compared to control synovial fibroblasts. In addition, synovial fibroblasts from MoM patients retained higher contractility and increased responsiveness to chemotaxis in matrix contraction. Their mechanical properties at a single cell level increased as observed by a 60% increase in contraction force and higher cell stiffness (3.3 kPa in MoM vs 2.18 kPa in control), as measured by traction force microscopy and atomic force microscopy. Further, fibroblasts from MoM patients promoted immune cell invasion by secreting monocyte chemoattractant protein 1 (MCP-1, CCL2) and induced monocyte differentiation, which could also be associated with excess accumulation of synovial macrophages.

**Conclusion:**

Synovial fibroblasts exposed in vivo to MoM THA implants that release CoCr wear debris displayed dramatic phenotypic alteration and functional changes. These findings unravelled an unexpected effect of the CoCr alloy and demonstrated an important role of synovial fibroblasts in the undesired tissue reactions caused by MoM THAs.

## Background

Soft tissue reactions develop in response to all total hip replacements, however between 2008 and 2015 in the UK, 92.5% of hip revision procedures involved metal-on-metal (MoM) total hip arthroplasty (THA) implants [1], manufactured from a cobalt chromium alloy (CoCr). The formation of pseudotumours, aseptic lymphocyte-dominated vasculitis-associated lesion (ALVAL) and metallosis (often defined as aseptic fibrosis and local necrosis) are believed to be the sequelae of large amounts of CoCr metal particles and dissociated ions released from the hip bearing surfaces due to both wear processes and corrosion [2–4]. Synovial tissues retrieved at the MoM revision surgery have shown the presence of a large number of amorphous particles predominantly composed of chromium and cobalt [5–7]. The majority of these particles from the implants are in the nano-sized scale and the severity of the adverse tissue reactions is reported to be related with the complexity of the particles and blood metal ion levels [7]. The spectrum of these tissue reactions is extensive and ranges from small asymptomatic cysts to destructive periprosthetic soft-tissue masses, which may lead to severe symptoms of pain and ultimately implant failure [8]. The integrity of the synovial lining is often impaired by inflammatory cell infiltrates (lymphocytes, macrophages, plasma cells and giant cells), which indicate the extent of ALVAL [9]. While immune cells play a key role in the initiation and progression of the tissue reactions [1, 10], some evidence suggests that chronic inflammation occurs because of the activated fibroblasts, resulting in the inappropriate survival and retention of immune cells within inflamed tissue [11]. However, the role of stromal fibroblasts and tissue/extracellular matrix (ECM) organisation in the inflammatory response following exposure to cobalt metal wear and corrosion products in vivo remains largely unknown.

Previously, we reported that metal ions, particularly cobalt, alter healthy human dermal fibroblasts in vitro by stimulating contraction and the release of pro-fibrotic signals from macrophages, thus leading to fibrotic reactions and matrix remodelling [12]. To further investigate whether this interaction between fibroblasts and immune cells also occurs in vivo following exposure to CoCr particulates and associated ions, we examined ex vivo the properties of fibroblasts isolated from periprosthetic synovial tissues from patients implanted with a MoM (manufactured from standard CoCr alloy) THA, retrieved during revision operations. To uncouple the role of any underlying inflammatory condition, we used a control group of synovial fibroblasts isolated from patients undergoing a primary hip replacement operation. As soft tissue reactions have not been reported as often in patients with either ceramic-on-polyethylene or metal-on-polyethylene THA bearings [1], fibroblasts from these patients were not investigated. We hypothesise that the synovial fibroblasts retrieved at revision operations would show altered mechanical and functional properties that could be related to the released wear and corrosion products from the bearing surfaces of the MoM THAs, and could thereby stimulate inflammatory responses in vitro. The implication of this study suggests that synovial fibroblasts have a role in the in vivo inflammatory soft tissue reactions following MoM THA.

## Materials and methods

### Sample collection

#### Ethics approval and patient selection

Ethics approval [07/Q0401/25, West London Research Ethics Committee] and written patient consent was obtained for the use of tissue samples (synovial membranes) removed during surgery. Tissue samples were collected from patients (Table 1) undergoing either primary hip replacement surgery (Primary THA) or a revision surgery where a primary MoM hip implant had been used (Revision THA). All tissue specimens were anonymised. Patients with unilateral and bilateral total hip replacements were included in this study, whilst patients with prostheses other than MoM, were excluded from this study. The criteria for failed MoM implants included unexplained pain, implant loosening and high cobalt and chromium ion levels in the blood, as described in Table 1. The original reason for surgery for the revision THAs would have been OA. The exclusion criteria in this study were infection, mechanical instability or prosthesis malalignment.

**Table 1:** Summary of patient demographics

	No.	Gender (M/F)	Age (year)	Time from primary to revision (year)	Reason for surgery
Primary THA	1	F	53	/	OA
	2	F	53	/	OA
	3	F	54	/	OA
	4	F	56	/	OA
	5	M	49	/	OA
	6	F	58	/	OA
	7	F	62	/	OA
	8	F	55	/	OA
Revision THA	1	F	85	10	Painful hip with elevated metal ions
	2	F	66	5	Painful hip
	3	M	63	14	Painful hip
	4	F	90	7	Trunionosis
	5	M	75	6	Painful hip with elevated metal ions
	6	M	75	11	Painful hip
	7	F	56	9	Aseptic loosening
	8	M	79	9	Elevated metal ions

(*OA* osteoarthritis)

#### Tissue harvest and storage

Primary fibroblasts were isolated from biopsies obtained from patients once the joint capsule was exposed during surgery. Another part of the tissues used for histology was stored in 10% Neutral Buffered Formalin (Sigma, UK) at room temperature before processing.

### Histology

Fixed tissues were dehydrated through a series of graded ethanol baths (70, 90%, absolute ethanol), cleared in a xylene bath (Sigma, UK) and finally embedded in paraffin and placed on ice at − 20 °C overnight. The tissue in the wax blocks was trimmed and cut into 5 μm sections (Accu-Cut® SRMTM 200 rotary microtome, Torrance, CA, USA). Slides were deparaffinised by washing in 2 changes of xylene and graded ethanol baths (absolute ethanol, 90, 70%). Antigen retrieval was performed to unmask the antigenic epitope of the tissue sample by boiling the deparaffinised sections in citrate buffer at pH 6.0. Endogenous peroxidase activity was blocked by incubating sections in 3% H_2_O_2_ solution (Sigma, UK) in PBS at room temperature for 10 min followed by 2 rinses in PBS. To reduce background staining the samples were incubated with normal goat serum (5% in PBS) to block nonspecific binding sites. After removing the blocking buffer, 100 μl of rabbit polyclonal anti-type-I collagen antibody (1:1000 in 1% goat serum in PBS, Abcam, UK) or rabbit polyclonal anti-α-smooth muscle actin (SMA) antibody (1:2000; Sigma-Aldrich, USA) was added to the sections on the slides and incubated in a humidified chamber at room temperature for 1 h, after which the slides were washed twice in PBS. 100 μl of a diluted biotinylated secondary antibody (in 1% goat serum in PBS, Vector Laboratories, UK) was then applied to the sections and incubated in a humidified chamber at room temperature for 30 min, washed in PBS, then incubated for 30 min with VECTASTAIN® ABC Reagent (Vector Laboratories, UK). Samples were washed and incubated in peroxidase substrate solution (Vector Laboratories, UK) with positive staining visualized by using a DAB Peroxidase (HRP) Substrate Kit (Vector laboratories, UK).

### Isolation and culture of synovial fibroblasts

Biopsy samples were collected in DMEM medium, then transported on ice, and primary fibroblasts were isolated within 6 h. The harvested tissues were transferred into a 10 cm tissue culture dish in DMEM medium using a sterile forceps and finely minced into approximately 1 mm^3^ pieces using sterile scalpels. The pieces of tissues were placed in a 15 ml centrifuge tube containing 10 ml of digestion solution (0.1% collagenase in DMEM medium, Sigma, UK) and incubated at 37 °C under rotation for 2 h. 15 ml DMEM medium was subsequently added to dilute the collagenase which was passed through a 70 μm cell strainer (BD Biosciences, UK) to obtain a single-cell suspension. The cell suspension was centrifuged for 5 min at 200 x g at room temperature, the supernatant was discarded and the pellet resuspended in DMEM cell culture medium (10% FBS and 1% P/S). Harvested cells were counted and seeded at approximately 4000 cells/cm^2^ in a T75 flask. The human synovial fibroblasts were cultured at 37 °C and 5% CO_2_, with a first medium change 24–48 h later. The medium was changed every 2–3 days.

### Cell metabolic activity measurement

Synovial fibroblast proliferation was measured using CellTiter 96® Aqueous One Solution Cell Proliferation Assay (Promega) according to the manufacturer’s instructions. Fibroblasts were cultured in 96-well tissue culture plates at 5 × 10^3^ cells/well over 72 h. Cell culture supernatants were aspirated and 100 μL of serum-free DMEM medium containing 10% CellTiter reagent was added to each well. Plates were incubated for 2 h at 37 °C and the absorbance was read at 490 nm using an Infinite F50 plate reader (Tecan, UK). Five replicates of each exposure were tested and the entire assay was repeated in three separate experiments. The cell viability was determined as a percentage of control cell viability.

### Collagen contraction

A contraction assay was used to measure collagen contraction by fibroblasts. Fibroblasts (1 × 10^4^ cells/ml) were first mixed with 1.5 mg/ml neutralized rat tail collagen solution (3 parts of 2 mg/ml collagen solution with 1 part DMEM medium). 0.5 ml fibroblast-containing collagen solution was added to 24-well cell culture plates and allowed to polymerize for 30 min at 37 °C. Then 0.5 ml of serum-free cell culture medium was added and the gels were incubated for 12 h. Following incubation, these gels containing fibroblasts were gently detached from the wells using fine straight and curved forceps. The medium was aspirated and replaced with DMEM cell culture media containing either 10% serum, PDGF-BB (10 ng/ml, Peprotech, UK) or TGF-β1 (5 ng/ml, Peprotech, UK). The gel contraction was recorded following 12 h of exposure to these media.

### Mechanical properties measurement

#### Traction force measurement

Briefly, 22 mm coverslips (Corning, USA) were treated with Hexamethyldisilazane (HMDS, VWR, UK), rinsed, air dried and a gel solution of 5% containing acrylamide (Biorad, UK), 0.25% bis-acrylamide (Biorad, UK), 0.05% ammonium persulfate (10% APS, Sigma), 0.1% 1, 2-Bis (dimethylamino) ethane (TEMED, Sigma, UK) and carboxylate-modified beads (fluorescent red, diameter 0.2 μm, 1% v/V, ThermoFisher) was applied. After polymerization, the gel was activated with heterobifunctional cross-linker 1 mg/ml Sulfo-SANPAH (Sigma) and thoroughly rinsed with distilled water. The gels were then coated with type I rat tail collagen (0.2 mg/ml) overnight. Fibroblasts were plated at a density of 3 × 10^4^ cells/well in a serum-free DMEM and incubated overnight at 37 °C and 5% CO_2_ in a humid incubator before imaging (LS720 Microscope, Lumascope, UK). Brightfield for cells and 525 nm for bead image stacks were acquired at each position. After imaging, fibroblasts were lysed with 1% SDS with the positions re-imaged. Five different cells from each of 8 control samples and 8 MoM samples were analysed. A custom-built analysis pipeline implemented in LabVIEW (National Instruments, Austin, TX) was used to measure the traction forces as previously described [13].

#### Atomic force microscopy (AFM)

The stiffness of fibroblasts was measured using Advanced Quantitative Imaging mode on a JPK NanoWizard 4 system in combination with an inverted microscope (Axio Observer Z1, Zeiss). Cantilevers (HYDRA6R, AppNano, UK) were calibrated by measuring the sensitivity against a stiff polystyrene substrate, and then fitting the resonance peak in the thermal noise spectrum to determine the spring constant (approximately 0.08 N/m). A ROI of 100 × 100 μm was selected to cover an entire cell. Indentations were performed in a format of 32 × 32 at a loading/unloading speed of 50 μm/s, which minimized cell movement during scanning without compromising the resolution. All the AFM experiments were carried out at 37 °C in FBS-free medium. Force-indentation curves were further processed involving background subtraction, height correction and fitting with a modified Hertzian model.

### Collagen production

To measure the hydroxyproline produced by fibroblasts, the culture medium was collected after 48 h of culture and hydrolyzed in 6 N HCl for 24 h at 105 °C. The amount of 4-hydroxy proline in the hydrolyzate was determined at a wavelength of 570 nm on a microplate reader (BMG Nova Star, BMG LABTECH) using the conventional colorimetric method [14].

### Chemokine profiling

The cell culture media was harvested after 48 h and processed for profiling chemokines using the Proteome Profiler Human Chemokine Array Kit (R&D system, Minneapolis, MN), which detects a panel of 31 chemokines (Table 2). The array membranes were reacted with the mixture of conditioned media and the antibody cocktail for 18 h at 4 °C. After 3 washes, they were briefly incubated with secondary antibodies conjugated with horseradish peroxidase (HRP). The membranes were then exposed to HRP substrate for 30 min. The intensity of the reaction was quantified using a BIO-RAD ChemiDoc MP Imaging System (Bio-Rad, UK); the pixel densities on developed X-ray film were analysed using Image Lab software (Bio-Rad, UK). The supernatants were assayed for MCP-1 concentrations with a human MCP-1 ELISA kit (Biolegend, UK).

**Table 2:**
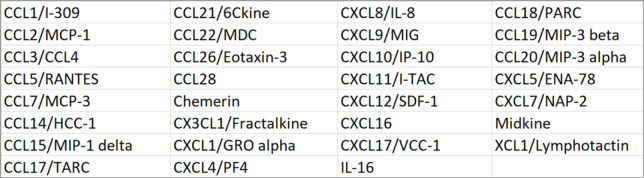
Chemokines detected with the Proteome Profiler Human Chemokine Array

### Transwell migration assay

Monocyte migration was investigated using 6.5 mm Transwell chambers with 8 μm pores (Costar, Corning, NY, USA). Briefly, 1 × 10^5^ U937 monocytes in serum-free DMEM medium were added to the upper chamber of an insert. The lower chamber was filled with cell culture supernatant from fibroblasts cultured for 48 h to encourage cell migration down the FBS chemotactic gradient. After 12 h, cells that transmigrated to the bottom well were collected and counted.

### Fluorescence-activated cell sorting (FACS) analysis

After 24 h of serum withdrawal, the percentage of apoptotic fibroblast were determined via FACS utilising a FITC Annexin V Apoptosis Detection Kit with PI (Biolegend, UK). The collected cell suspensions were transferred to FACS tubes (BD Biosciences; Oxford, UK), centrifuged for 5 min at 350 x g and the supernatant aspirated. Cell suspensions were washed twice with cold cell staining buffer (Biolegend, UK), and then resuspended in Annexin V Binding Buffer at a concentration of 0.5 × 10^7^ cells/ml. 100 μl of cell suspension was transferred to a 5 ml test tube and 5 μl of FITC Annexin V with 10 μl of Propidium Iodide Solution was added. The mixture of cells and staining reagent was allowed to incubate for 15 min at room temperature in the dark prior to analysis with flow cytometry. Stained cells were washed and re-suspended in 200 μl of PBS and analysed using a BD ACCURI C 6 flow cytometer (Biosciences, USA) and FlowJo software (TreeStar, USA).

### Co-culture of U937 monocytes and synovial fibroblasts

Porous plasma-treated polycarbonate inserts (pore size: 0.4 μm, Transwell, Corning) were used for non-contact co-culture of fibroblasts and U937 monocytes. MoM and control synovial fibroblasts were added to the top of the Transwell® insert at 2.5 × 10^4^cells/well and U937 monocytes were cultured at 5 × 10^4^cells/well in 24-well tissue culture plates providing a co-culture within each well. The increase in the differentiation of U937 monocytes was quantified by counting the cells that floating in the medium collected from each well of tissue culture plates by 0 h, 12 h and 24 h of co-culture.

### Image analysis

Images were acquired on an upright microscope (Nikon Eclipse). For image quantification, the mean percentage area positive for 10 randomly selected high powered fields (× 10 magnification) was calculated using ImageJ analysis software as described previously [15].

### Data analysis and statistics

All data were expressed as the mean + SEM of at least three independent experiments. The statistical differences between groups were analysed with Student t-test (SPSS Inc., Chicago, IL, USA). A value of *p* < 0.05 was considered statistically significant.

## Results

### Tissue remodelling of patients with metal-on-metal hip implants

**Fig. 1:**
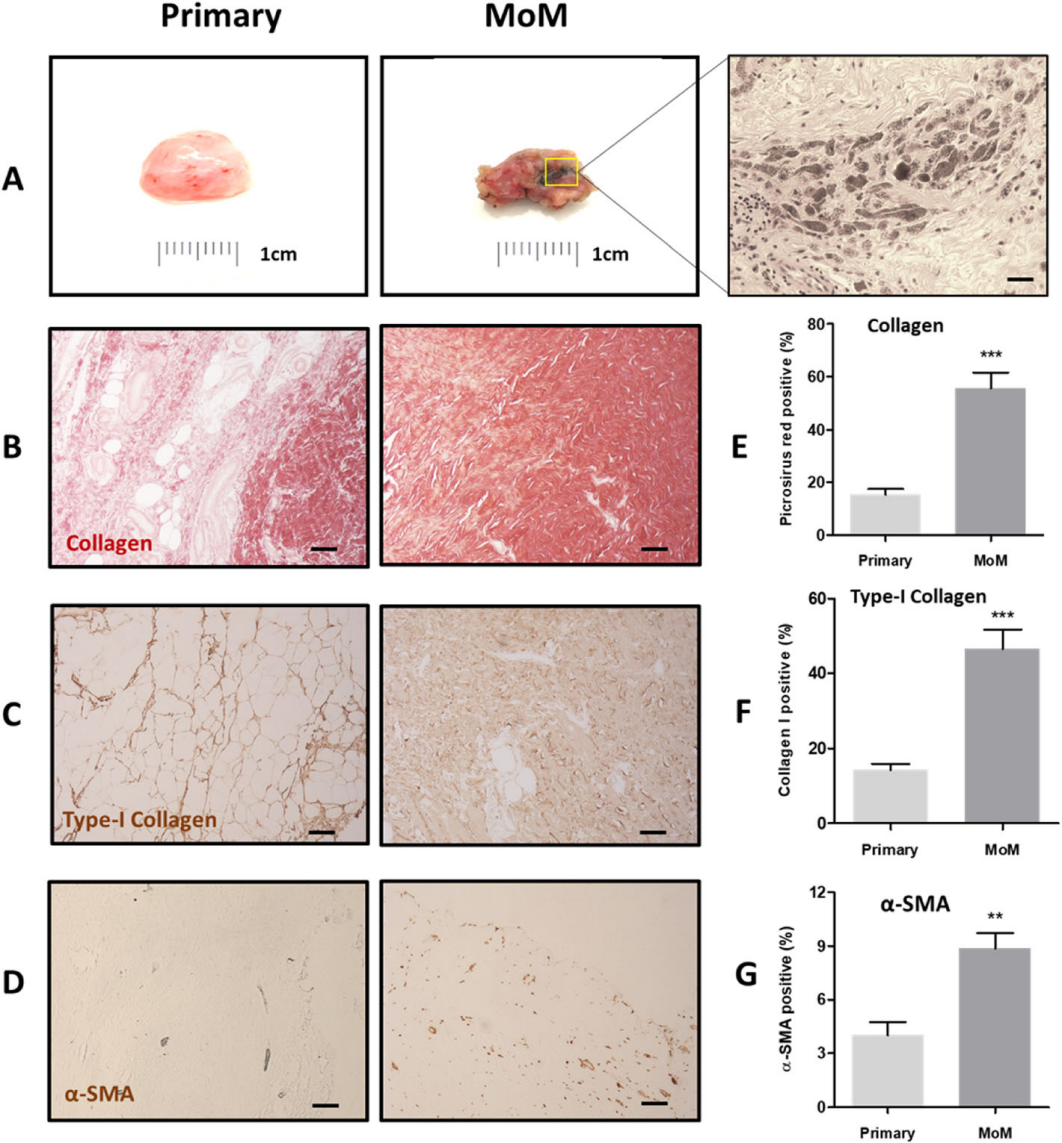
Synovial tissues from primary THA patients and patients with metal-on-metal THA implants. **a** Macroscopic evaluation of synovial membrane isolated from primary and revision MoM THA implants. Scale bar indicates 50 μm. **b** Collagen visualized by picrosirius red and **c** collagen I visualized by immunohistochemistry staining with quantification showed in **e** and **f**. **d** α-SMA positive fibroblasts in synovial membrane tissue with quantification shown in **g**. Scale bar indicates 200 μm in (**b**-**d**). Data was obtained from 8 primary THA and 8 MoM tissue samples. For each slide/patient, 10 random fields were imaged for analysis. Bars represent mean + SEM; unpaired t-test, ** *p* < 0.01, *** *p* < 0.001

A gross morphological change to the periprosthetic tissue was observed in tissues collected from patients with failed MoM implants. The biopsies were generally denser, pigmented and contained fibrotic regions (Fig. 1a). At the microscopic level metal wear or corrosion products in some of the MoM cases were observed, which appeared a dark-grey or green morphology (Fig. 1a) as previously reported [16]. To determine the level of a fibrotic change, we assessed the collagen deposition. The control tissues obtained at primary THA operations were characterised by a wide distribution of fat cells (adipocytes) and approximately 20% collagen content as determined by Picrosirius Red staining (Fig. 1b,e) and more specifically collagen type I (Fig. 1c,f). Elevated levels of collagen were observed in the MoM tissues, with approximately 55% positive staining for collagen and 45% for collagen type I. We further observed a significant increase in the number of myofibroblasts in the MoM tissues from less than 4% to nearly 9% (Fig. 1d,g) as determined by the positive staining for α -SMA.

### MoM fibroblast are more resistant to apoptosis

**Fig. 2:**
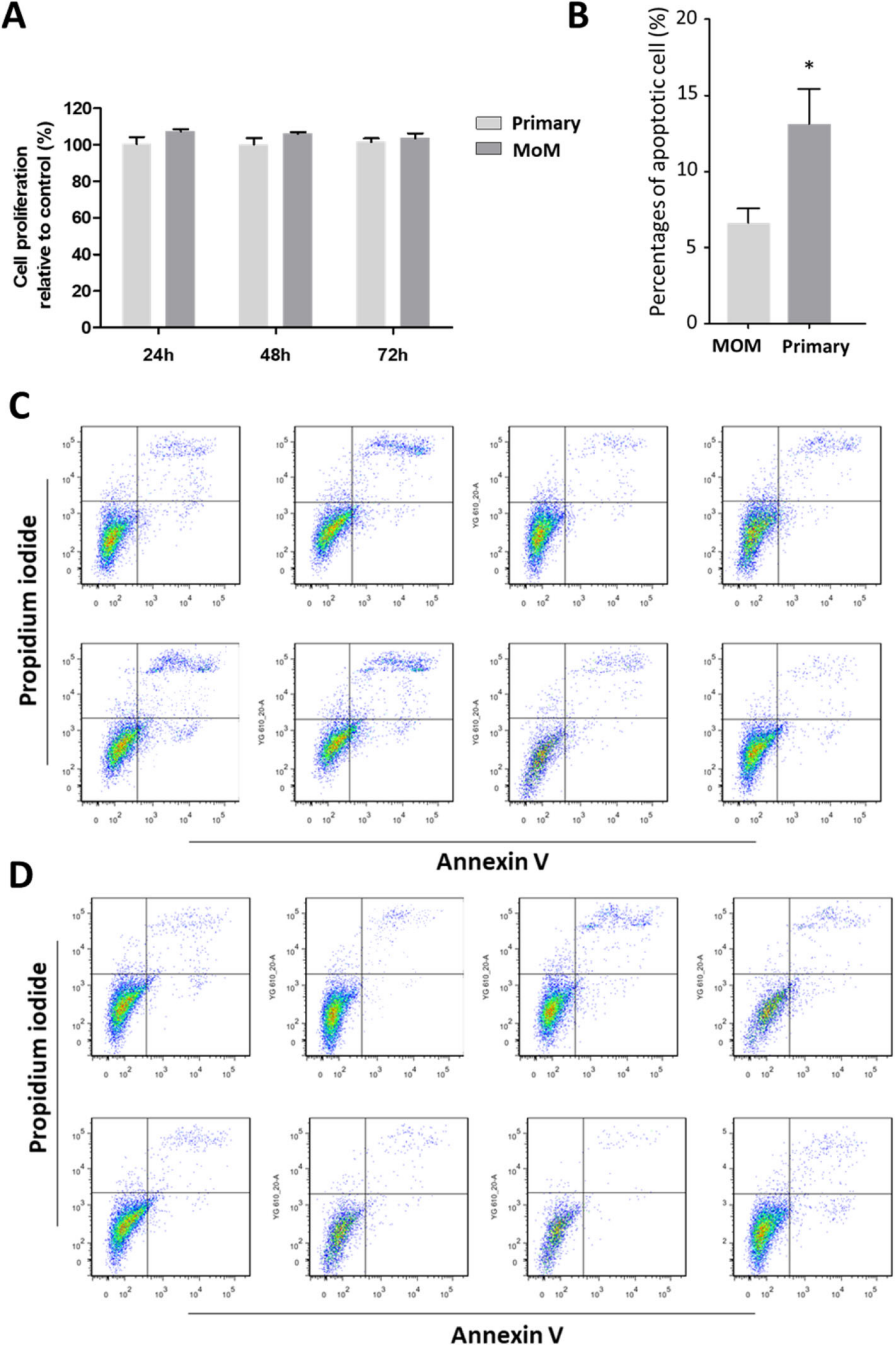
Proliferation and apoptosis of primary and MoM fibroblast. **a** Proliferation of primary and MoM fibroblasts over 72 h measured by MTS assay. Apoptosis of primary and MoM fibroblast induced by serum withdrawal. **b** Quantification of apoptotic cells of primary and MoM fibroblasts. Data was from 3 independent experiments using fibroblasts from each primary and MOM patient; unpaired Student’s t-test; *p < 0.05. Representative cell apoptosis results of primary (**c**) and MoM (**d**) fibroblasts from each patient assessed by Annexin V-FITC/PI assay via FACS

The balance between cell proliferation and apoptosis plays a key role in tissue homeostasis. First, we investigated whether the increased matrix deposition in tissue is due to an increase in proliferative capacity of synovial fibroblasts exposed to metal products from MoM implants. We found that ex vivo MoM fibroblasts showed the same proliferative capacity as control synovial fibroblasts over 72 h of culture (Fig. 2a). It has been reported that fibroblast survival could be altered under certain pathological condition in a manner of reduced cell apoptosis, which contributes to the development and maintenance of fibrosis [17]. Although no significant difference in the proliferative ability was identified between primary and MoM fibroblasts, MoM synovial fibroblasts showed reduced apoptosis rates following serum withdrawal (Fig. 2b) compared with the control cells. Primary (Fig. 2c) and MoM fibroblast (Fig. 2d) survival was determined by serum withdrawal-induced apoptosis, analysed by an Annexin V-FITC/PI assay via FACS. According to the results shown, after 24 h of serum withdrawal, the percentage of apoptotic cells for MoM fibroblasts were approximately 6%, compared to nearly 14% for the control fibroblasts. These data suggest that MoM fibroblasts are more resistant to apoptosis, which may partially explain their accumulation and tissue remodelling in the patients with CoCr alloy implants.

### Matrix remodelling by human synovial fibroblasts

**Fig. 3:**
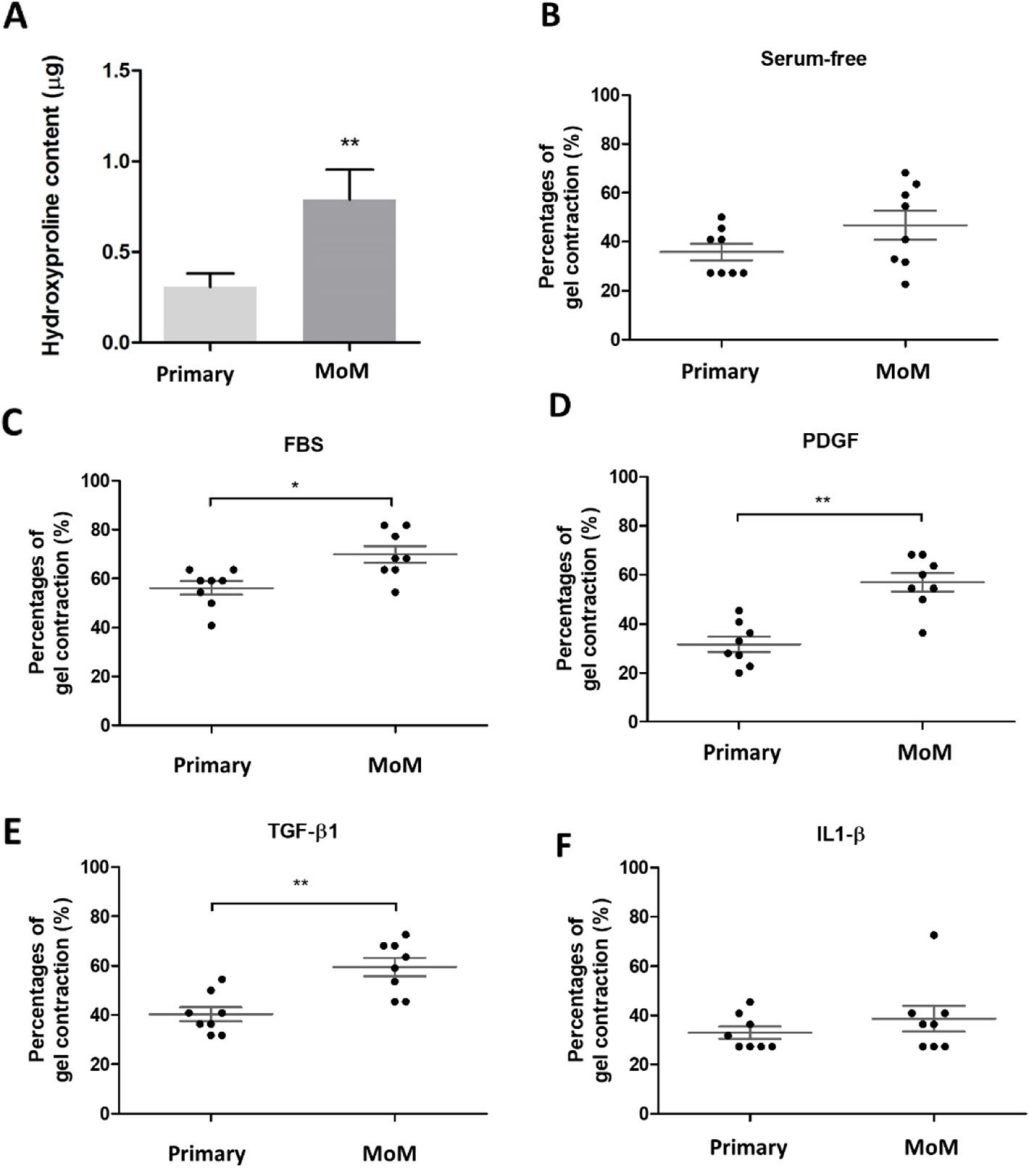
Synovial fibroblasts from patients with MoM implant display increased ECM matrix production and contractility. **a** Collagen released by cultured synovial fibroblasts into the cell culture medium after 48 h. Bars represent mean + SEM. **b**-**f** Fibroblasts from MoM and primary THA were embedded in a collagen matrix and contraction was measured after 48 h, in serum-free medium (**b**), medium with 10% serum (**c**) or with cytokines (10 μg/ml PDGF (**d**), 5 μg/ml TGF-β1 (**e**) and 10 ng/mL IL-1β (**f**). Shown is mean ± SEM for gel contraction after 48 h of culture. Each individual data point is the average of 3 independent experiments using fibroblasts from each patient. * *p* < 0.05, ** *p* < 0.01

To elucidate the in vivo tissue alterations in tissue density and matrix deposition, the isolated synovial fibroblasts were examined ex vivo for matrix production. We measured the hydroxyproline content as a surrogate for collagen. A statistically significant increase in production of pro-collagen by MoM synovial fibroblasts was observed when compared to the control synovial fibroblasts from primary THAs (Fig. 3a). This suggests that synovial fibroblasts isolated from tissues surrounding a MoM implant which requires revision, but has been in use, releasing metal and ionic debris for 5–14 years, have enhanced matrix production ability and retain that capability ex vivo in the absence of the metal stimulation. To investigate whether the MoM synovial fibroblasts have greater capability to deform matrix, which is an important aspect of pro-fibrotic alteration, their contractility potential was analysed. An in vitro collagen gel contraction assay was performed in various conditions, including serum-free conditions and in the presence of serum (10% FBS), PDGF (10 ng/mL), TGF-β1 (5 ng/mL) and IL-1β (10 ng/mL). In the serum-free condition, the contractility of MoM and control synovial fibroblasts was comparable (Fig. 3b), while in the presence of serum the contractility of MoM synovial fibroblasts was significantly increased (*p* < 0.05). When mimicking inflammatory conditions, by adding PDGF or TGFβ, the MoM fibroblast contractility further increased to 56.9 ± 3.8% (from 31.7 ± 3.1%) in the presence of PDGF (*p* < 0.01) and to 59.5 ± 3.7% (from 40.3 ± 2.9%) in the presence of TGF-β (p < 0.01) (Fig. 2c-e). While IL-1β have not obvious effects on the contractility of both MoM and control synovial fibroblasts. These results suggest that MoM synovial fibroblasts displayed a distinctive bio-mechanical response to associated inflammatory signalling.

### MoM-induced alterations of fibroblast mechanical properties

**Fig. 4:**
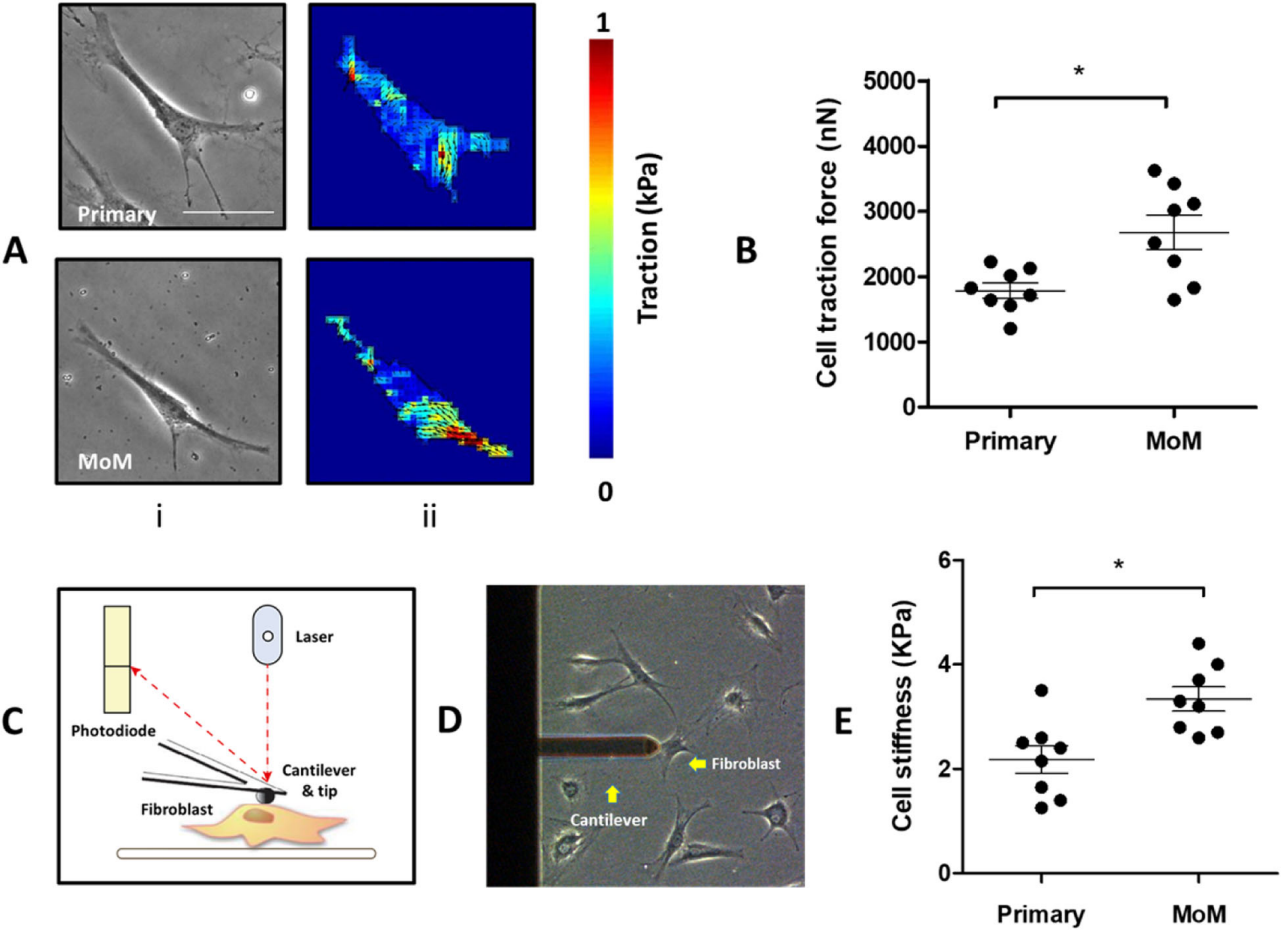
Contractile forces and cell stiffness of fibroblasts from patients during primary THA and at revision from a MoM THA. **a** Bright field images of individual cell (i) and corresponding stress magnitude maps (ii); **b** Quantification of cellular contractile force of fibroblasts from the cells of individual groups; *n* = 40 for each group (each data point represents the mean value of 5 cells analysed for each patient). **c** Schematic of AFM setup for measuring cell stiffness. **d** Image showing the AFM cantilever and synovial fibroblasts. **e** Quantification of cell stiffness for primary and MoM fibroblasts; n = 40 (each data point represents the mean value of 5 cells analysed for each patient); unpaired Student’s t-test. *p < 0.05

The contractility of cells is regulated by their mechanical properties. Previously, we showed that cobalt exposure affects cell elastic modulus and contractile force of human dermal fibroblasts [12]. To study the link between the increased contractility of MoM synovial fibroblasts and alterations in their mechanical properties, we performed Traction Force Microscopy and Atomic Force Microscopy. First, we measured the traction forces of resting synovial fibroblast (following 24 h of serum starvation). A significant increase, of nearly 60%, in cellular traction forces (*p* < 0.05) was observed in MoM synovial fibroblasts when compared to the control (Fig. 4a and b). Cell stiffness is closely associated with cell behaviour and the alterations in cell traction forces could be coupled with alterations in cell elastic modulus, which has been suggested as an indicator of cytoskeleton rearrangement [18]. We observed that the cell elastic modulus measured with AFM (Fig. 4c and d) was also significantly higher (3.33 ± 0.65 KPa) in MoM synovial fibroblasts compared with the control (2.18 ± 0.74 KPa) as shown in Fig. 4e.

### MoM fibroblasts promote immune cell invasion and differentiation

**Fig. 5:**
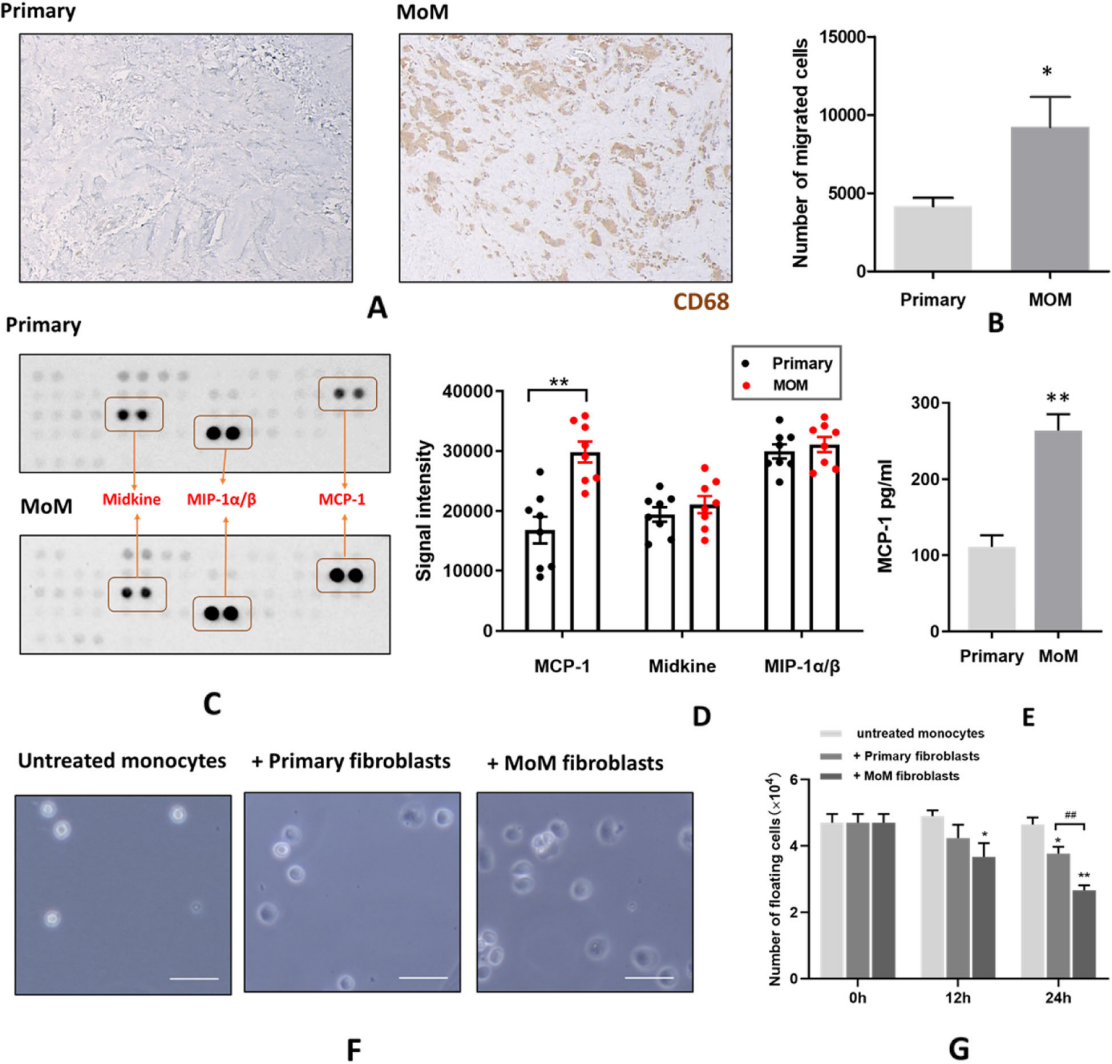
MoM fibroblast recruit and promote monocytes differentiation. **a** Immuno-histochemical staining of macrophage infiltration in synovial tissue from a MoM patient (magnification × 100). **b** Migrated monocytes in harvested medium from primary and MoM fibroblasts. **c** Chemokines detected using Human Chemokine Array. **d** Signal intensity of measurable chemokines of from (**c**) showing mean ± SEM. **e** Quantification of MCP-1 secreted in the cell culture medium, showing mean + SEM. Data was from 3 independent experiments using fibroblasts from all primary and MoM patients; unpaired Student’s t-test; *p < 0.05, **p < 0.01. **f** Images indicated that co-culture with synovial fibroblast promoted monocyte differentiation into adherent macrophages. Scale bar = 10 μm. **g** The increase in monocyte differentiation was quantified by counting the floating cells. Data shown as mean + SEM; *n* = 3; unpaired Student’s t-test; *p < 0.05, **^/##^*p* < 0.01

As reported in the previous section, growth factors released by immune cells drive the contractile activity of MoM synovial fibroblasts. One of the key immune cells activated by cobalt released from CoCr implants (MoM) are CD68^+^ macrophages, which release pro-fibrotic signals and enhance the fibrotic response of fibroblasts [4]. The histological analysis of the tissue biopsies showed a significant increase in CD68^+^ macrophages in MoM tissues (Fig. 5a). However, whether the MoM synovial fibroblast release pro-inflammatory signals to recruit macrophages, is unclear. To study the pro-inflammatory properties of MoM synovial fibroblasts, a migration assay was performed. The U937 monocyte migration was significantly enhanced in the presence of cell culture supernatants from MoM fibroblasts (Fig. 5b). By examining the chemokine profile of the supernatants (Fig. 5c), a significant elevation in MCP-1 (monocyte chemoattractant protein 1) was observed in the MoM fibroblast cell culture supernatant compared to the control (Fig. 5d&e). This suggests that MoM fibroblasts could potentiate the tissue immune response through the secretion of MCP-1, thereby enhancing monocyte infiltration. Also, to test the effect of MoM fibroblasts on monocyte differentiation, fibroblasts from MoM and control patients were co-cultured with U937 human monocytes. It could be noticed that both MoM and control synovial fibroblasts induced monocyte differentiation into macrophages, as indicated by increased cellular adhesion to the substrate with the number of floating U937 monocytes were significantly lower compared to the untreated cells at the 24 h time points (Fig. 5g). Furthermore, the number of floating U937 monocytes decreased to a greater extent in the presence of MoM synovial fibroblasts compared with control fibroblasts (Fig. 5g). Therefore, these results indicated the presence of higher amount of pro-inflammatory factors from MoM fibroblasts that could promote the invasion and differentiation of human monocyte.

## Discussion

Despite being one of the most significant issues in the field of total hip arthroplasty, the precise mechanisms associated with adverse tissue reactions to wear debris generated in patients following the implantation of a MoM implant (which generates CoCr particles and dissociated ions in use) responsible for the devastating failure of these implants, remains poorly understood. Many cell lines have previously been used to investigate the effects of CoCr wear particles and ions in vitro including macrophages, lymphocytes and osteoblasts. In vitro studies have revealed that CoCr metallic debris and ions trigger the release of an array of proinflammatory mediators from macrophages [19–21], such as transforming growth factor α (TGF-α), macrophage colony-stimulating factor (M-CSF), platelet-derived growth factor (PDGF) and epidermal growth factor (EGF). Cobalt has also been known to promote intracellular reactive oxygen species formation that lead to a wide range of deleterious effects and irreversible damage.

Fibroblasts are one of the central participants for wound healing and inflammation. They play a positive role in promoting the recovery of functional tissue but equally are capable of driving it towards chronic inflammation, fibrosis or even tissue necrosis depending on their exposure to biochemical cues [19]. It has also been reported that fibroblasts, one of the most active tissue resident mesenchymal cells, may display site or disease-specific phenotypes after exposure to inflammatory signals, which could induce a dysregulated homeostatic balance of extracellular environment [22]. This motivated the present study, in which in vitro assays were applied to characterise some of the key features of primary human synovial fibroblasts and investigate their contribution to the tissue response and disease progression.

The findings of this study demonstrated that synovial tissues from patients undergoing revision surgery due to the failure of the MoM device were macroscopically and histologically different from tissues isolated from patients undergoing primary THA surgery, with evidence of extensive tissue remodelling characterised by replacement of adipocytes with dense areas of collagen (Fig. 1a-c). In addition, we observed enhanced activation of synovial fibroblast activation with a significant increase in the number of myofibroblasts in the tissues (Fig. 1d). Fibroblasts are the primary cell type responsible for ECM proteins synthesis to build up the connective tissue. Moreover, they promote ECM remodelling via transmitting force to contract and compact the surrounding matrices to achieve tensional homoeostasis, which have a significant impact on other cells’ behaviour [23]. Our data demonstrated that stimulated fibroblasts isolated from patients undergoing a MoM THA revision were more efficient at contracting collagen matrix and were also more responsive to biochemical stimuli involving cytokines and growth factors (Fig. 3). They were also found to display altered cell force responses compared to the control primary cells (Fig. 4). Disruption of the balance between cell proliferation and apoptosis could affect tissue homeostasis. The enhanced survival of MoM fibroblasts when exposed to apoptotic signal (Fig. 2) also confirmed the alteration of fibroblasts phenotype and undesired cellular response after exposure to CoCr metal wear and corrosion products.

Previous research has largely focused on the cytotoxic effects of CoCr alloy products on fibroblasts ex vivo, with little attention being paid to their influence on local tissue homeostasis in vivo [24–26]. Indeed recent evidence has suggested that the deposition of ECM, produced by fibroblasts as well as tissue mechanics play a vital role in the regulation of the inflammatory process [27]. For instance, stiffer tissue substrates may enhance immune cell invasion and the release of pro-inflammatory cytokines by macrophages [28, 29]. In addition to their structural role in shaping the microenvironment, fibroblasts also modulate immune responses via their production of cytokines and chemokines [23].

Periprosthetic synovial tissues from patients with MoM THA implants at the revision operation, were characterized by intensive immune cells infiltration. However, the key stimulus of driving the immune cell invasion is largely unknown. MCP-1/CCL2 is one of the critical chemokines that mediates migration and infiltration of monocytes and macrophages during inflammatory response. In vivo and in vitro studies suggest that MCP-1 plays a direct role in the development of many fibrotic lesion by affecting fibroblasts behaviour [30–32]. In this study, we found that fibroblasts surrounding failed MoM THA revision patients may play an indispensable role in the recruitment of immune cells by secreting MCP-1, which enhances chemotactic migration of monocytes and exacerbate inflammatory reactions (Fig. 5). Our results also indicated that MoM fibroblasts produce stimulating factors to promote the differentiation of monocytes, thus potentiating the pathogenic persistence and retention of macrophages in the synovial tissues surrounding MoM implants. Recent studies have reported non-MoM THAs, such as metal-on-polyethylene (MoP), to be presenting adverse tissue responses similar to the MoM THAs. An adverse response to MoP implants, such as the formation of pseudotumours, may still be linked to the release of cobalt ions from the metal head [33–35], albeit at a lower level. This suggests that any wear/corrosion debris containing cobalt (nano) particulates or ions, may have a detrimental influence on the hip tissue microenvironment.

Overall, we have shown that synovial fibroblasts exposed to MoM THAs in vivo which require revision*,* undergo phenotypic alteration which is associated with dramatic functional changes that can be observed ex vivo. The response to an inflammatory lesion involves a complex interplay of diverse cellular and tissue elements that restrain tissue invasion and ultimately establish normal tissue integrity [36]. Instead of being a passive player in the immune system, fibroblasts actively define the organisation of tissue microenvironments and modulate cell activities by conditioning the local cellular and cytokine microenvironment [22, 37]. The inappropriate activation and accumulation of fibroblasts stimulated by wear debris from failing MoM implants could prevent the resolution of acute inflammation thereby leading to chronic, persistent inflammation.

## Conclusions

**Fig. 6:**
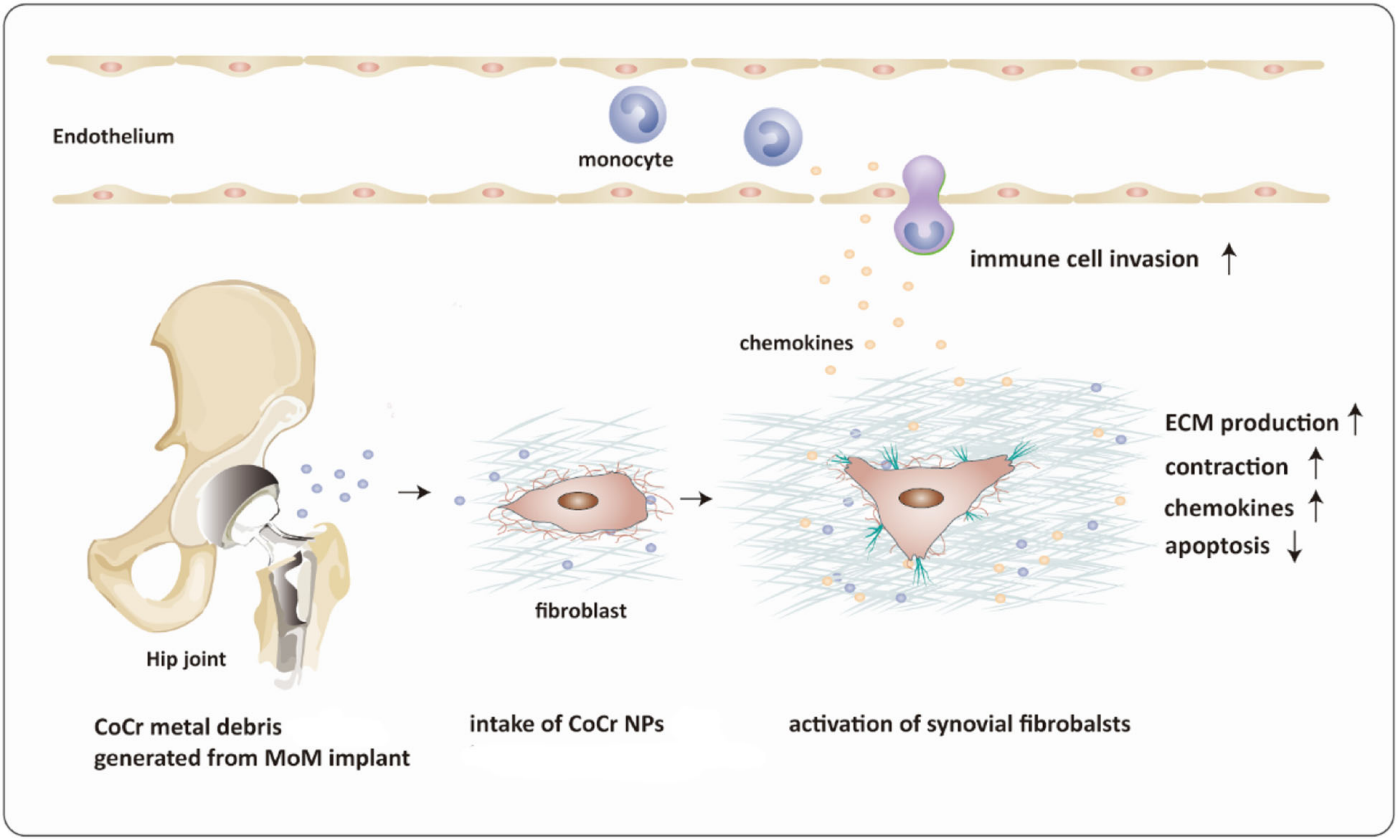
Schema for the distinctive effects of CoCr particles on fibroblast phenotype and their interplay with immune cells in the patients with CoCr based implants

This study unravels the distinctive influence of MoM total hip arthroplasties, which release CoCr wear and corrosion debris in the form of particles and related ions, on the fibroblast phenotype and their interplay with immune cells (Fig. 6). Synovial fibroblasts exposed to MoM THAs in vivo displayed dramatic phenotypic alteration and functional changes. These findings suggest direct in vivo impact of failing MoM THAs on the synovial fibroblasts and local tissue homeostasis.

## Data Availability

Data supporting the findings are found within the manuscript and supplemental material.
